# Consistent Clustering Pattern of Prokaryotic Genes Based on Base Frequency at the Second Codon Position and its Association with Functional Category Preference

**DOI:** 10.1007/s12539-021-00493-w

**Published:** 2021-11-24

**Authors:** Yan-Ting Jin, Cong Ma, Xin Wang, Shu-Xuan Wang, Kai-Yue Zhang, Wen-Xin Zheng, Zixin Deng, Ju Wang, Feng-Biao Guo

**Affiliations:** 1 School of Life Science and Technology, University of Electronic Science and Technology of China, Chengdu, 611731 China; 2 Key Laboratory of Combinatorial Biosynthesis and Drug Discovery, Ministry of Education and School of Pharmaceutical Sciences, Wuhan University, Wuhan, 430071 China; 3 School of Biomedical Engineering, Capital Medical University in Beijing, Beijing, 100069 China; 4 School of Biomedical Engineering, Tianjin Medical University, Tianjin, 300070 China

**Keywords:** Base frequency, A_2_ versus T_2_, The second codon position, Two unequal clusters, Protein function preference

## Abstract

In 2002, our research group observed a gene clustering pattern based on the base frequency of A versus T at the second codon position in the genome of *Vibrio cholera* and found that the functional category distribution of genes in the two clusters was different. With the availability of a large number of sequenced genomes, we performed a systematic investigation of A_2_–T_2_ distribution and found that 2694 out of 2764 prokaryotic genomes have an optimal clustering number of two, indicating a consistent pattern. Analysis of the functional categories of the coding genes in each cluster in 1483 prokaryotic genomes indicated, that 99.33% of the genomes exhibited a significant difference (*p* < 0.01) in function distribution between the two clusters_._ Specifically, functional category P was overrepresented in the small cluster of 98.65% of genomes, whereas categories J, K, and L were overrepresented in the larger cluster of over 98.52% of genomes. Lineage analysis uncovered that these preferences appear consistently across all phyla. Overall, our work revealed an almost universal clustering pattern based on the relative frequency of A_2_ versus T_2_ and its role in functional category preference. These findings will promote the understanding of the rationality of theoretical prediction of functional classes of genes from their nucleotide sequences and how protein function is determined by DNA sequence.

**Graphical abstract:**

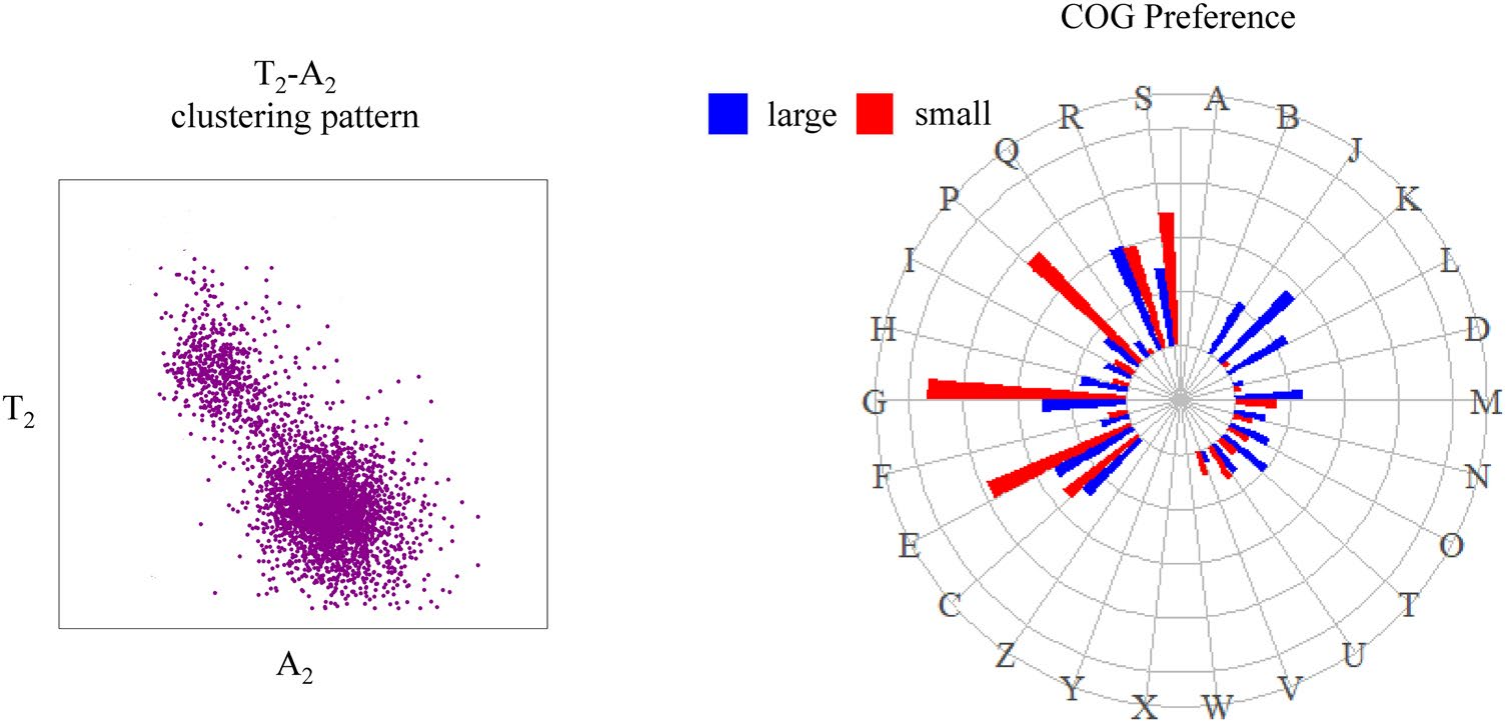

**Supplementary Information:**

The online version contains supplementary material available at 10.1007/s12539-021-00493-w.

## Introduction

The genetic code is a set of rules that defines how the four-letter code of DNA is translated into the 20-letter code of protein [1, 2]. Being employed in all organisms, the genetic code has many conserved and universal features, including successive triplets without overlapping [3], degeneracy for mutation tolerance [4] and codon usage bias that is meaningful for the regulation of gene expression [5–10]. Studies of codon and base usage can facilitate our understanding of the origin and evolution of genetic code. The choice of nucleotide at a specific codon position in coding genes has received much attention. For example, researchers have found strong C base preference at the second position of the second codons in the cell envelope-related genes [11], and clarified that the physical origins of codon positions strongly influence cotranslational protein folding [12]. For the broadest studies are of the third codon position. Codon degeneracy mainly manifests at this position and among the synonymous codons, the one matching the most abundant tRNA usually has the highest frequency [13]. This coupling pattern has been thought to benefit translation efficiency [14]. Recent genomic-scale expression data analyses have demonstrated the global effect of synonymous codon usage bias on transcription and translation efficiency [15–19]. Synonymous codon usage can also regulate protein folding type [20–23] and mutation at the synonymous site may cause intolerance and disease in human [24]. In addition, codon position specific nucleotide bias has been employed by some computational tools as an important feature for the identification of functional genes [25–28].

Although the nucleotide bias of the three codon positions has been widely used in gene identification, few studies have connected the codon position-specific nucleotide pattern with gene function distribution, particularly in the second codon position. In 2002, we observed an interesting pattern of base usage of coding genes in *Vibrio cholerae*. All coding genes could be divided into two unequal clusters according to the relative base frequencies of A and T at the second codon position, and the coding genes in the two clusters exhibited significant difference in protein functions [29]. We hypothesized that this pattern might appear widely in the prokaryotic domain and that it could be connected with gene function. Hence, a larger scale analysis was needed to validate our speculation.

In this paper, a systematic analysis of thousands of genomes across three domains revealed that the clustering phenomenon according to the base frequencies of A and T at the second codon position is almost universal and is especially remarkable for prokaryotes. Further study on 1483 prokaryotes with COG (clusters of orthologous groups) annotation [30, 31] shows that 99.33% of genomes have a significant difference (*p* < 0.01, by Chi-squared test) in the functional distributions of genes between the two unequal clusters. Furthermore, the overrepresent categories in the two clusters were consistent among species and prokaryotic phyla. Here, we revealed a consistent of A_2_–T_2_ associated clustering pattern and consistent functional influence in prokaryotes.

## Materials and Detailed Methods

### Genome Data Collection

The prokaryotic genomic data used in this study were downloaded from NCBI (ftp://ftp.ncbi.nlm.nih.gov/genomes/archive/old_refseq/Bacteria/). Three files, i.e., ‘all.ffn.tar.gz’, ‘all.faa.tar.gz’ and ‘all.ptt.tar.gz’ were retrieved (on March 26, 2017), and contained FASTA files for nucleotide coding regions, FASTA for amino acids, and protein tables of all the prokaryotes available. After removing the sequences for plasmids and fragments from every genome, we obtained 2764 prokaryotic genomes with the sequences of protein coding genes (164 archaea and 2600 bacteria) for further analysis. The 1035 eukaryotic genomic data were downloaded from Ensembl (https://asia.ensembl.org/downloads.html), the details are shown in Table 1. Altogether, the gene sequences and annotation information of 3799 genomes from three domains of life were collected (Table 1).

**Table 1:** The protein coding genes of 3799 genomes collected for analysis

Domains	Genomes	Detail
Prokaryotes	2764	164 archaea and 2600 bacteria
Eukaryotes	1035	68 metazoa, 186 protists, 735 fungi, 44 plants, 1 *M. musculus* and 1 *H. Sapiens*
All genomes	3799	

### *K*-Means Algorithm

*K*-means is a statistical method for partitioning observations in a data set into a given number of clusters (*K*). In this study, the *K*-means was used to divide the coding genes in a genome into a specific number of clusters (e.g., *K* = 2) based on the relative base frequencies of A and T at the second codon position. We wanted to determine whether there was a consistent clustering pattern based on A_2_–T_2_ in the currently available genomes.

There are many basic clustering techniques, which can be classified into five categories: partitioning methods, density-based methods, grid-based methods, hierarchical methods and model-based methods [32]. The *K*-means algorithm is a partitioning method. It can give a definite number of final clusters and furthermore is highly efficient to implement. Here we need to cluster the genes of thousands of prokaryotic genomes hence we choose this method.

### Silhouette Coefficient Analysis

As an unsupervised machine learning algorithm, *K*-means can be used to group the protein coding genes in a genome into *K* clusters (*K* = 2–9 in our case). We further adopted silhouette coefficient analysis to determine the optimal number of clusters into which the genes could be divided. Briefly, the silhouette coefficient was used to quantify the separation between the resulting clusters by measuring how close each gene in one cluster is to genes in the neighboring clusters. In this study, we used the silhouette coefficient to evaluate the performance of *K* in the range from 2 to 9 for all genomes.

### Clusters of Orthologous Groups (COGs) Function Annotation

Comparison of proteins encoded in numbers of complete genomes from many major phylogenetic lineages and elucidation of consistent patterns of sequence similarities allows the delineation of many clusters of orthologous groups (COGs) [31]. There are 26 function categories (Table 2) in this framework and each category is denoted with a specific letter. Among all the prokaryotic genomes analyzed, 1483 had complete COG annotation.

**Table 2:** The 26 function categories could be classed into four super-categories

Super-category	Number	Code letter
Information storage and processing	5	J, K, L, A, B
Cellular processes and signaling	11	D, Y, V, T, M, N, Z, W, U, O, X
Metabolism has eight categories	8	C, G, E, F, H, I, P, Q
Poorly characterized	2	R, S

### Chi-Squared Test

We further used the Chi-squared (χ2) test to evaluate whether there was a significant difference in the COG function categories in the two unequal clusters obtained by *K*-means clustering. For each genome, the χ2 test involved a 2 × 26 Chi-square table. The first row contained the number of genes in each COG function category in the large cluster, and the second row contained the number of genes in each COG function category in the small cluster. The differences were significant for 1475 out of the 1483 genomes (*p* < 0.05) and highly significant for 1473 genomes (*p* < 0.01).

### Measurement of the Difference in Specific Functional Category in the Two Clusters

1 $$\begin{gathered} F\left( {P_{{{\text{small}}}} } \right) = \frac{{N\left( {P_{{{\text{small}}}} } \right)}}{{N\left( {{\text{small}}} \right)}} \hfill \\ F\left( {P_{{{\text{large}}}} } \right) = \frac{{N\left( {P_{{{\text{large}}}} } \right)}}{{N\left( {{\text{large}}} \right)}}, \hfill \\ \end{gathered}$$

2 $$\begin{array}{*{20}c} {{\text{P}}\;{\text{category}}} & {{\text{smaller}}} & {{\text{larger}}} \\ {F\left( {P_{{{\text{small}}}} } \right) > F\left( {P_{{{\text{large}}}} } \right)} & 1 & 0 \\ {F\left( {P_{{{\text{small}}}} } \right) < F\left( {P_{{{\text{large}}}} } \right)} & 0 & 1 \\ {F\left( {P_{{{\text{small}}}} } \right) = F\left( {P_{{{\text{large}}}} } \right)} & 0 & 0 \\ \end{array} ,$$

Equations (1), (2): (1) *N*(*P*_small_): the number of coding genes belonging to the P functional category in the small cluster. *N*(small): the total number of coding genes in the small cluster. *F*(*P*_small_): the ratio of *N*(*P*_small_) to *N*(small) and so for *F*(*P*_large_). (2) Significance is determined by whether the difference is beyond 5% of the lower proportion value.

### Bacteria Taxonomy

TaxonKit (https://github.com/shenwei356/taxonkit) [33] was used to rapidly assign the prokaryotic genomes into different phyla, and we retained only those phyla containing more than 10 genomes for further research.

## Experimental Results

### Grouping Protein Coding Genes by Base Frequency at the Second Codon Position

To analyze the base distribution at the three codon positions, *f*(*X*_*n*_) was defined to represent the frequency of a base at a certain codon position for a coding gene, with ‘X’ denoting bases A, T, C or G, and ‘n’ denoting the 1st, 2nd or 3rd codon position. For example, *f*(*A*_2_) represents the frequency of base A at the second codon position of a coding gene. We directly employed base T in coding DNA instead of base U in the counterpart mRNA for convenience. When the distribution of *f*(*X*_*n*_) was checked separately, no universal pattern was observed, although several frequencies, e.g., *f*(*X*_1_), *f*(*X*_3_), *f*(*C*_2_) and *f*(*G*_2_), showed skewed distributions for a few genomes. However, when the combination of *f*(*A*_2_) and *f*(*T*_2_) was applied, we found that, the protein coding genes gathered into two unequal clusters in the scatter plot in most genomes, similar to what we observed previously in *Vibrio cholerae* [29].

**Fig. 1:**
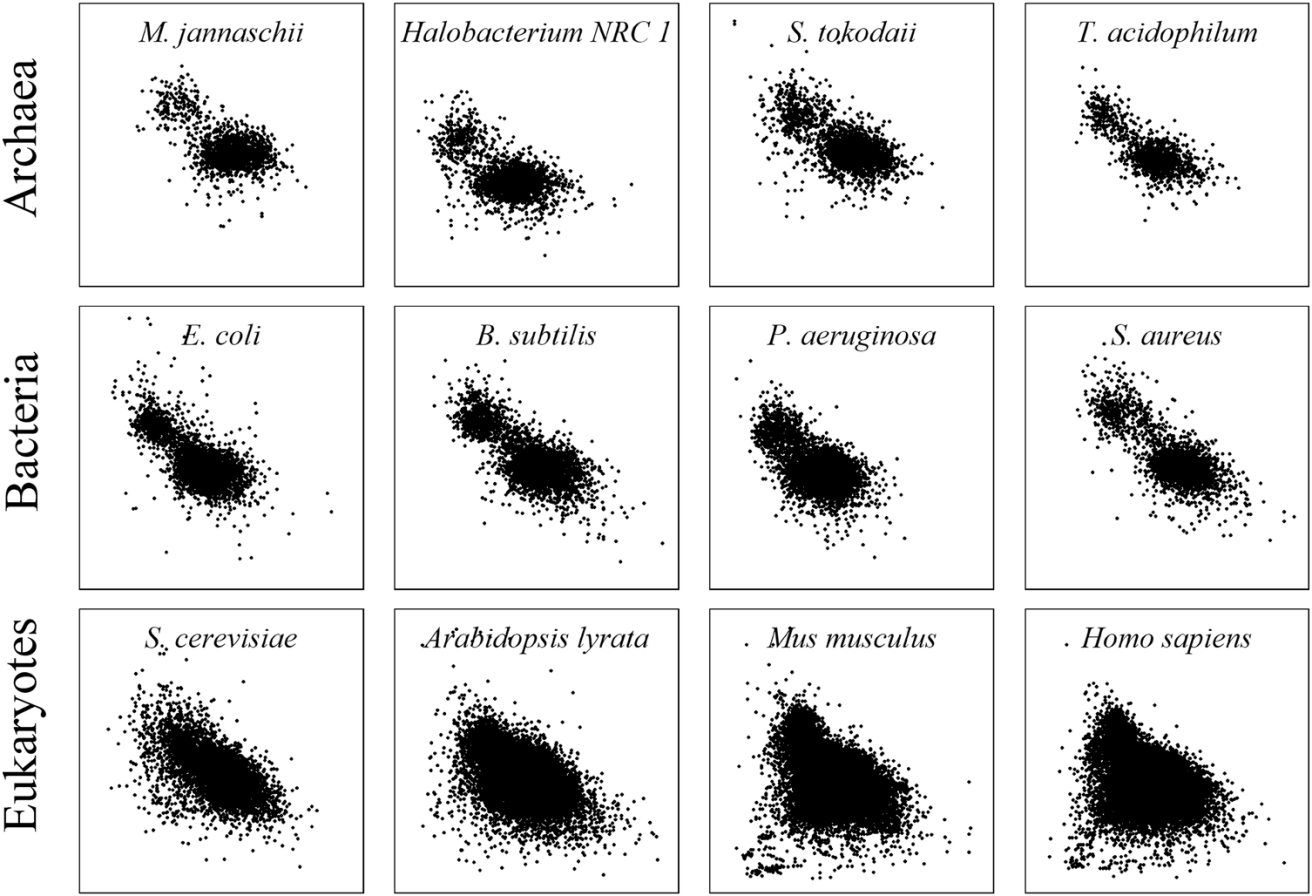
Coding genes are divided into two unequal clusters by the base frequencies of A and T at the second position of codons. The scatter plots of 12 representative genomes from three domains with *f*(*A*_2_) as the *x* axis and *f*(*T*_2_) as the *y* axis both ranging from 0 to 0.7. The clustering phenomenon in archaea and bacteria is significant: a small cluster with much higher *f*(*T*_2_) and a large cluster with similar *f*(*T*_2_) and *f*(*A*_2_). This phenomenon was not significant in eukaryotes

Then, a systematic survey of *f*(*A*_2_) and *f*(*T*_2_) was conducted across the three domains of life (164 archaea, 2600 bacteria and 1035 eukaryotes), and it was shown that the protein coding genes in a genome could be divided into two unequal clusters. The smaller cluster has a larger *f*(*T*_2_) and smaller *f*(*A*_2_), and the larger cluster has a relatively smaller *f*(*T*_2_) and larger *f*(*A*_2_). Such a pattern was evident and almost universal in archaea and bacteria. This pattern could also be observed in eukaryotes but was usually nonsignificant (Fig. 1).

### Silhouette Coefficient to Measure the Optimal Cluster Number

**Fig. 2:**
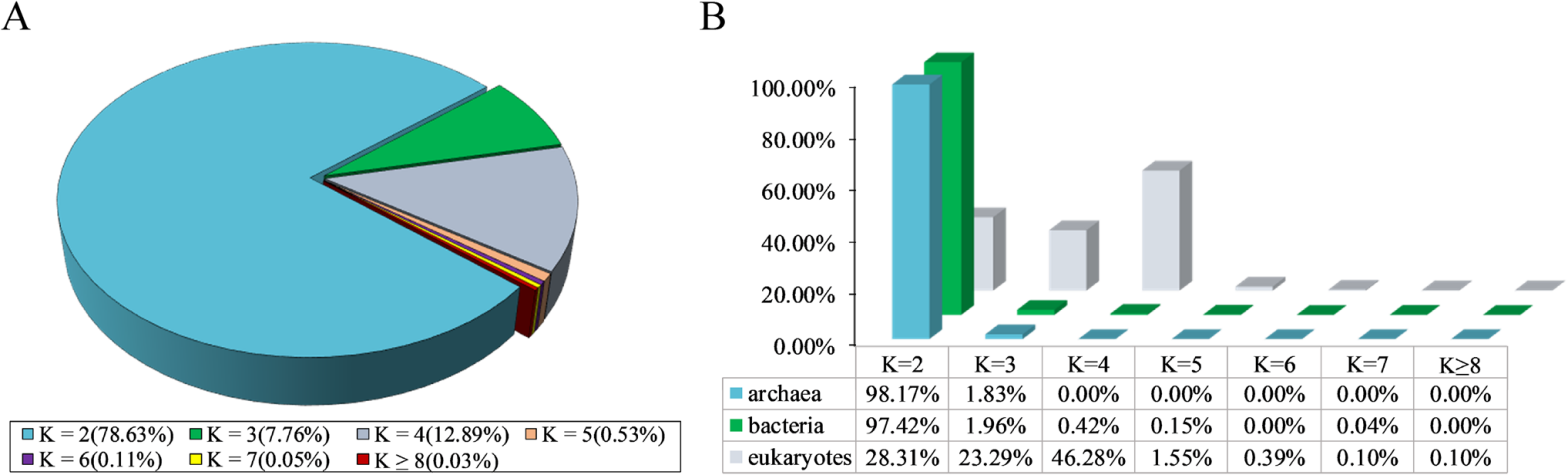
The best choice of cluster number is 2. **A** Taking all three domains as a whole, 78.63% of genomes had an optimal *K* of 2. **B** General distribution of quantitative optimal *K* values indicates that two clusters are the best choice for prokaryotes and some of eukaryotes (Table S1)

To check whether the genes in the genomes could be significantly divided into two clusters based on the *f*(*A*_2_) and *f*(*T*_2_), we adopted the quantitative method of *K*-means clustering to divide the genes in each genome into different groups. Then, the silhouette coefficient was used to measure how many clusters the genes could be divided into with the maximum intercluster distance and minimum intracluster distance. We surveyed 2764 prokaryotic genomes and 1035 eukaryotic genomes. We found that 98.17% (161/164) of the archaeal genomes and 97.42% (2533/2600) of bacterial genomes could be optimally divided into two clusters (Fig. 2; Table S1).

Although the tendency of gene clustering could still be observed for eukaryotes, particularly higher eukaryotes (multicellular organisms), the best clustering number varied widely. Of all the eukaryotes examined, only 28.31% had an optimal clustering number of two, while optimal cluster numbers of three and four were found for 23.29% and 46.28% of the genomes, respectively (Fig. 2B). This phenomenon might be associated with the complexity of multicellular eukaryotes, which have many more genes in their genomes, with gene functions closely related to factors such as cell type and transcription regulation and also requiring more elaborate cell structures and intricate metabolic networks [34]. For simplicity, we focused on the prokaryotic genomes in the following analyses.

### Biased Functional Distribution of Genes in the Two Clusters

We further checked the function of the genes in the two clusters divided by *f*(*A*_2_) and *f*(*T*_2_). Of the 2764 prokaryotic genomes, COG (cluster of orthologous groups) annotations could be retrieved for 1483. When the Chi-squared test was applied to the 26 COG functional categories, a significant difference (*p* < 0.01) was detected for the functions of genes included in the two clusters for 99.33% of the genomes examined (Table 3; Table S2).

**Table 3:** The Chi-squared test results of 1483 genomes on the protein function difference in the two unequal clusters

	*p* ≥ 0.05	*p* < 0.05	*p* ≥ 0.01	*p* < 0.01
Genome number	8	1475	10	1473
Frequency	0.54%	99.46%	0.67%	99.33%

### Differences in the Distribution of Protein Function Categories in the Two Unequal Clusters

**Fig. 3:**
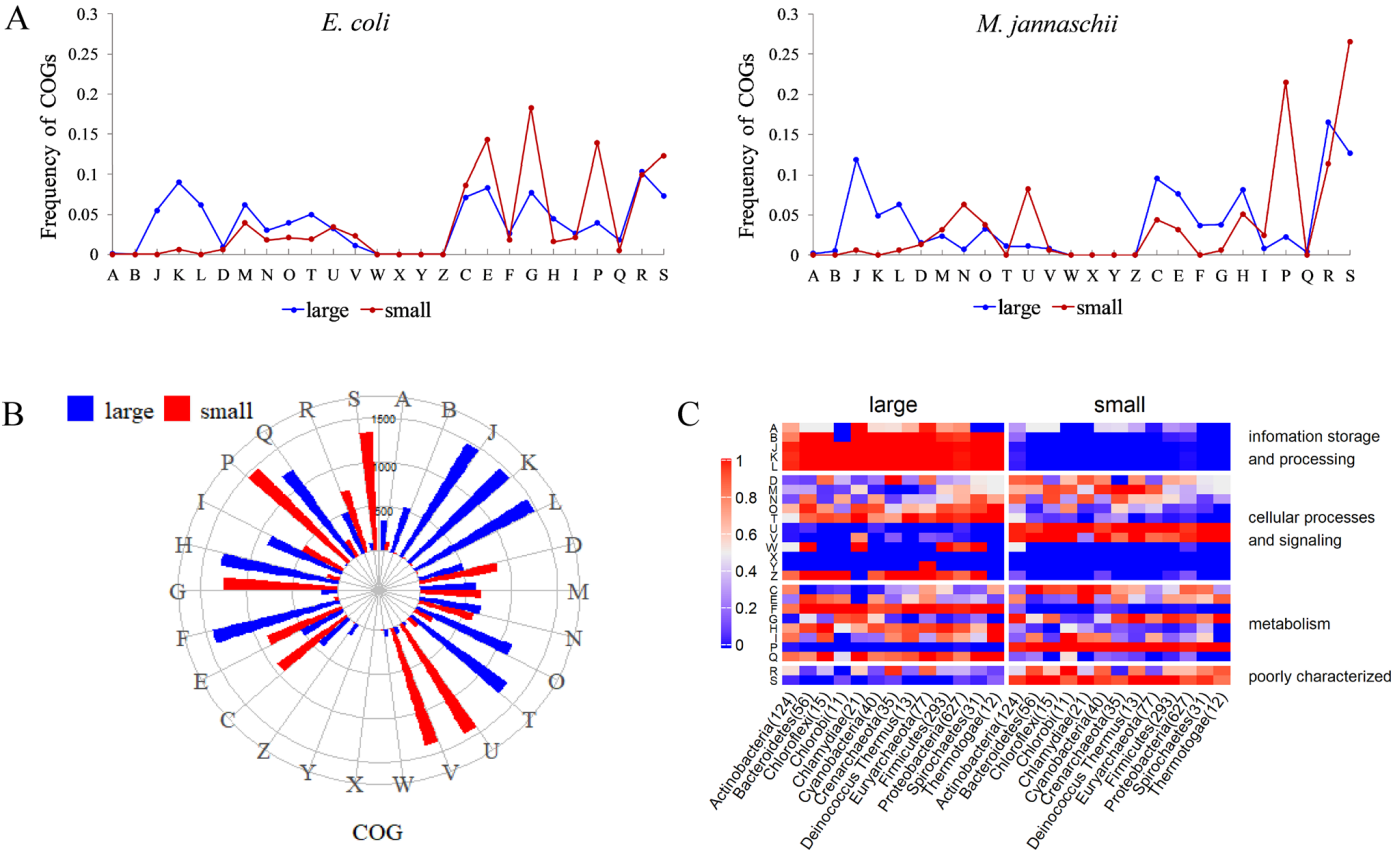
The distribution and difference in COG functional categories in the two unequal clusters of 1483 genomes. **A** In two representative genomes in prokaryotes, P-related genes prevailed in the small cluster, while J-, K- and L-related genes were observed at a higher proportion in the large cluster. **B** Cumulative overrepresented genome numbers of 26 functional categories. The 26 functional categories are listed clockwise, beginning with A and ending with S, according to super-category: information genes, cellular processes and signaling, metabolism and poorly characterized genes. **C** Overrepresentation in large or small clusters for each functional category at the phylum level

To see a clearer pattern of the functional difference in genes in the two clusters, *E. coli* and *M. jannaschii* were chosen as a representative of bacteria and archaea, respectively (Table S3). Since the larger cluster contained much more genes than the smaller cluster, we calculated the proportion of genes belonging to every functional category in each cluster through Eq. (1). By comparing the gene function distributions in the two clusters, we found that gene related to inorganic ion transport and metabolism (COG category code P) were much more prevalent in the smaller cluster than in the larger cluster. In contrast, genes related to translation, ribosomal structure and biogenesis (J), transcription (K) and replication, recombination and repair (L) were more likely to be included in the larger cluster (Fig. 3A). This pattern seemed to be universal as it was observed in all the genomes checked.

Then we used Eq. (2) to examine whether a given category is overrepresented in the larger (A_2_ preference) or smaller (T_2_ preference) cluster. Using the P category as an example, for a certain genome, if *F*(*P*_small_) > *F*(*P*_large_) and the difference was greater than 5%, for P, we assigned a value of 1 to the smaller cluster and 0 to the larger cluster. In contrast, if *F*(*P*_small_) < *F*(*P*_large_) and the difference was greater than 5%, the smaller cluster was assigned a value of 0, while the larger cluster was assigned a value of 1. If the difference between *F*(*P*_small_) and *F*(*P*_large_) was less than the 5% threshold, we defined both clusters as 0, denoting that no significant difference existed in the P category between the smaller and the larger clusters. Using this method**,** we were able to determine how many genomes exhibited overrepresentation of each functional category in the two clusters. Among the 26 COG categories, P, U, V, S, and G were overrepresented in the smaller cluster for 98.65% (1463/1483), 95.75% (1420/1483), 92.92% (1378/1483), 90.42% (1341/1483), and 84.49% (1253/1483) genomes, respectively. Comparatively, J, K, L, F, H, T, and Q were overrepresented in the larger cluster for 98.92% (1467/1483), 98.52% (1461/1483), 98.79% (1465/1483), 95.95% (1423/1483), 87.93% (1304/1483), 87.59% (1299/1483) and 81.66% (1211/1483) genomes, respectively (Fig. 3B; Table S4).

Since prokaryotes are classified into different phyla, we checked whether all the phyla had consistent preference of functional category. The 13 prokaryotic phyla, each containing more than 10 genomes (Table S4), were extracted for further lineage analysis. Each functional category was calculated for each genome through Eq. (2). Then we calculated the cumulative number of the overrepresented categories in each phylum. *Proteobacteria*, for example, contained 627 genomes, 528 of which had smaller cluster as 1 and 97 of which had larger cluster as 1 for C category [2 genomes had similar *F*(*C*_small_) and *F*(*C*_large_)]. Hence the overrepresented ratios in the small and large clusters in *Proteobacteria* were 528/625 (84.48%) and 97/625 (15.52%), respectively. If the number of genomes was zero in some GOG functional categories, the ratio of both the smaller and the larger clusters was defined as zero. In such cases, the sum of both the smaller and the larger cluster ratios would be 0. However, for most phyla, the sum of the ratios was 1.

In Fig. 3C, we focus on those categories with different colors in the left panel and right panel because two clusters have distinct ratios. For the left panel of the larger cluster, if one functional category has a consistent color that means all phyla have consistent preference and similar for the right panel of the smaller cluster. Following this rule, we found that J, K, L, F, H, and Q were consistently overrepresented in the larger cluster of all phyla, while P, U, and S were in the smaller cluster of all phyla. Therefore, at the species level (Fig. 3A), prokaryote level (Fig. 3B) and phylum level (Fig. 3C), a consistent preference of functional category was observed, i.e., J, K and L were significantly overrepresented in the larger cluster, whereas P showed the opposite pattern. Several other categories exhibited bias to a lesser degree.

## Discussions

Researchers have observed several clustering patterns in sequenced genomes. Médigue et al., analyzed codon usage in 780 *E. coli* genes [35]. Using factorial correspondence analysis, they illustrated that these genes could be divided into three classes. The first two classes are associated with expression level and the third is associated with mobility characteristics [35]. A similar pattern was found in the protein coding genes of *Bacillus subtilis* [36]. Since then, numerous studies have confirmed codon usage associated patterns in various prokaryotic genomes [37–39]. Ma and Chen defined the most deviated codon position (MDCP) and found that basing on MDCP, the CDSs of a genome can be classified into two clusters: typical and atypical [40]. Genes can also be divided into two separate clusters based on strand associated nucleotide bias [41–43]. All these clustering patterns are based on the distribution bias of codon usage, that is, nucleotide frequencies at three codon positions. Comparatively, here our clustering pattern is associated only with the second codon position and appears similarly in almost all prokaryotes.

On the other hand, codon usage has been used to cluster coding sequences of *Arabidopsis thaliana* genes in order to improve gene prediction [44]. Amino acid composition has been combined with machine-learning method to predict protein functional families and achieved accuracy of 69.1–96.1% [45]. Although DNA sequence could be also extracted as features to prediction protein function [46], in most cases features of amino acid frequency are adopted [47]. Here, we illustrated one example of direct link between nucleotide frequency and protein function categories. Therefore, our work would help to understand why protein function could be predicted from gene sequence. We hope future researches could get highly reliable prediction of protein function from DNA sequence and we think their used features would mainly associate with the second codon position, particularly A_2_ versus T_2_ frequency and in that sense our result would be well validated.

## Conclusion

By a systematic analysis of the base frequencies at the second codon position across the three domains of life, we found that the protein coding genes of prokaryotes can be divided into two unequal clusters based on *f*(*A*_2_) and *f*(*T*_2_). Further analysis showed significant difference in the proportions of genes belonging to certain COG categories in the two clusters. P-related genes were more prevalent in the smaller cluster, while J-, K- and L-related genes were more likely to be included in the larger cluster. Lineage analysis revealed that the bias was basically consistent among different phyla. Hence, this work demonstrates an almost universal clustering pattern by the frequency of T_2_ versus A_2_ and its basically consistent influence on functional category distribution among prokaryotic phyla. These findings can help us understand why coding potentiality and functional category assignment could be theoretically predicted from gene sequences.

## Supplementary Information

Below is the link to the electronic supplementary material.

Supplementary file1 (XLSX 105 kb)

Supplementary file2 (XLSX 464 kb)

Supplementary file3 (XLSX 1748 kb)

Supplementary file4 (XLSX 103 kb)
